# Phenotypical and Functional Polymorphism of Liver Resident Macrophages

**DOI:** 10.3390/cells8091032

**Published:** 2019-09-05

**Authors:** Andrey V. Elchaninov, Timur Kh. Fatkhudinov, Polina A. Vishnyakova, Anastasia V. Lokhonina, Gennady T. Sukhikh

**Affiliations:** 1 National Medical Research Center for Obstetrics, Gynecology and Perinatology Named after Academician V.I. Kulakov of Ministry of Healthcare of Russian Federation, 4 Oparina Street, Moscow 117997, Russia (A.V.E.) (P.A.V.) (A.V.L.) (G.T.S.); 2 Histology, Embryology and Cytology Department, Ministry of Healthcare of The Russian Federation, Pirogov Russian National Research Medical University, 1 Ostrovitianov Street, Moscow 117997, Russia; 3 Histology, Embryology and Cytology Department, Peoples’ Friendship University of Russia, 6 Miklukho-Maklaya Street, Moscow 117198, Russia; 4 Scientific Research Institute of Human Morphology, 3 Tsurupa Street, Moscow 117418, Russia

**Keywords:** monocytes, macrophages, Kupffer cells

## Abstract

Liver diseases are one of the main causes of mortality. In this regard, the development of new ways of reparative processes stimulation is relevant. Macrophages play a leading role in the regulation of liver homeostasis in physiological conditions and in pathology. In this regard, the development of new liver treatment methods is impossible without taking into account this cell population. Resident macrophages of the liver, Kupffer cells, represent a unique cell population, first of all, due to their development. Most of the liver macrophages belong to the self-sustaining macrophage cell population, whose origin is not bone marrow. In addition, Kupffer cells are involved in such processes as regulation of hepatocyte proliferation and apoptosis, remodeling of the intercellular matrix, lipid metabolism, protective function, etc. Such a broad spectrum of liver macrophage functions indicates their high functional plasticity. The review summarizes recent data on the development, phenotypic and functional plasticity, and participation in the reparative processes of liver macrophages: resident macrophages (Kupffer cells) and bone marrow-derived macrophages.

## 1. Introduction

Liver pathology still accounts a significant portion in the structure of morbidity [1,2]. In this regard, a constant search for new therapeutic approaches, including the usage of cellular and gene technologies, is needed [3,4]. Resident macrophages of the liver, Kupffer cells, which account 80–90% of all macrophages of the mammalian organism [5], are considered as one of the possible application targets of regenerative medicine [6]. In addition, Kupffer cells play a key role in maintaining liver homeostasis in normal and pathological conditions [7]. Macrophages serve as significant regulators of liver regeneration [8,9], development of inflammatory processes, fibrosis [10,11], cholestatic diseases [12], tumor progression [13], and liver damage during transplantation [14].

Development of new methods of treating liver diseases is impossible without studying the population of liver macrophages. However, this is an extremely difficult task, as macrophages represent a highly heterogeneous cell population. In this regard, the review focuses on current data on ontogenesis, phenotypic and functional plasticity of liver macrophages and their role in reparative processes.

## 2. The Sources of Macrophage Development

In accordance with modern concepts, macrophages in mammalian ontogenesis develop from three sources that correspond to three generations of hematopoietic stem cells [15,16,17].

The first generation emerges within the wall of the yolk sac; importantly, these first hematopoietic cells differ in origin from the endothelium of capillaries of the hematopoietic islets [16,17]. Presumably, these first hematopoietic progenitor cells give rise to microglia of the central nervous system [18,19]. Differentiation of microglia is distinguished by a number of features [20]. The conventional macrophage differons involve the stage of monocytes that circulate in the blood; in the differentiation of microglia, this stage is missing. The microglial precursors migrate directly to the central nervous system, where they mature to the definitive state [18,19].

The second generation of hematopoietic cells, erythro-myeloid progenitor cells, is derived from the hematogenic endothelium of the yolk sac capillaries; these cells subsequently colonize the embryonic liver. Macrophages that originate from these precursors are very similar to macrophages of the first generation by their molecular markers; however, their maturation involves the monocyte stage [16,17,18,19].

Another generation of hematopoietic cells derived from aorto-gonado-mesonephral zone endothelium. These cells populate the liver and the red bone marrow. Almost all organs of the embryo are colonized by macrophages of this generation, with the only exception being the central nervous system [18,19]. During embryogenesis fetal organs predominantly contain macrophages from the second and third generations while in postnatal period in most organs, the percentage of macrophages which descend from erythro-myeloid cells of the yolk sac gradually declines and the percentage of macrophages derived from the third-generation hematopoietic cells rises [15,17,21] However, several organic systems stand out. Central nervous system evidently is colonized only by the first generation macrophages. The liver and the epidermis are normally populated by the macrophages derived from the second generation of hematopoietic cells and got names – Kupffer cells and Langerhans cells, respectively [16,17].

Further in the course of development, within the skin dermis and the connective tissue of the intestinal mucous membranes, macrophages of the embryonic origin (the second generation) completely die out and are replaced by cells of the red bone marrow origin [15,16,17,22]. Developmental advantages and grounds for such dynamic distribution of macrophages within the body of mammals are obscure.

## 3. Macrophage Population of the Liver

As has been mentioned above, in the normal liver of mammals, the population of macrophages is composed largely of cells that originate from the erythro-myeloid progenitor cells of the yolk sac and are commonly referred to as Kupffer cells. Macrophages derived from the blood monocytes represent a minor population in the liver; according to different estimates, they constitute from 5% to 30% of total liver macrophages [15,23,24,25].

Such a large variation in quantitative estimation of the population of bone marrow-derived macrophages in the liver is due to specific choices of the markers used to identify them [26]. In our opinion, the most relevant choice is represented by a combination of Ly6C and CX3CR1 markers [15,17,25].

The majority of studies on macrophage ontogenesis were performed using laboratory mice. The postnatal mouse macrophages of bone marrow origin carry the surface protein Ly6C, which is absent from the surface of Kupffer cells [25,27]. Similarly, the protein marker CX3CR1 is used to recognize bone marrow-derived macrophages in rats (since Ly6C protein is not detected in rats) [25]. According to the data obtained with these markers, the proportion of bone marrow-derived macrophages in the liver constitutes approximately 5% [15,17,25]. Such an assessment is consistent with the observation that liver macrophages are diverse by their size and location. Two types of liver macrophages have been identified: large, located near the sinusoidal capillaries, and small, located around the central veins and portal tracts. Both of them express CD68; however, CD163 is expressed at a high level only by large Kupffer cells [28,29]. The small macrophages constitute about 8%, which is similar to the proportion of bone marrow-derived liver macrophages determined by Ly6C immunostaining [15,25]. These findings allow the possibility to consider small liver macrophages as the cells of monocytic origin.

The most controversial data is obtained using markers CD11b and CD68. These markers are expressed in a variety of cell types including all leukocytes; in addition, CD68 is found in endothelial cells and fibroblasts [30]. CD11b and CD68 participate in cell adhesion, cell migration, and phagocytosis; accordingly, their expression may undergo considerable rapid changes [30,31,32].

CD11b has been employed as a marker of bone marrow-derived macrophages in some studies [23,24,33]. It has been shown that, although all macrophages found in the liver (Kupffer cells and bone marrow-derived macrophages) express CD11b, the bone marrow-derived macrophages express it at a higher level [17,19,25,34,35].

Using the latest Single Cell RNA sequencing method in the human liver MacParland and colleagues established the presence of two populations of resident macrophages with pro-inflammatory and immunoregulatory (non-inflammatory) phenotypes. One of the main differences between these two populations was the expression level of the MARCO marker, which prevailed in non-inflammatory macrophages; MARCO+ macrophages were localized in the periportal zone [36]. Another study with Single cell RNA sequencing in a mouse steatohepatitis model also revealed two macrophage populations: Kupffer cells expressing Clec4f and monocytes-derived macrophages expressing Lyz2 [37]. Moreover, the population of macrophages with monocytic origin, found in the liver with steatohepatitis, was also extremely heterogeneous. The authors distinguish at least three subtypes of such macrophages: MoMF I showed a high expression of the extracellular matrix protein fibronectin 1 (Fn1), as well as microsomal glutathione S-transferase 1 (Mgst1) and methionine sulfoxide reductase B1 (Msrb1). MoMF II expressed few marker genes including Chil1, mainly indicating a bone marrow origin. MoMF III was characterized by a pronounced expression of Il1b [37].

In summary, according to one set of reports, the resident mouse liver macrophages have Ly6C^−^CX3CR1^low^ phenotype, whereas under the inflammatory conditions the liver tissue becomes infiltrated by the bone marrow-derived macrophages with Ly6C^+^CX3CR1^hi^ phenotype [25,38,39]. Other studies indicate that Kupffer cells are either F4/80^+^CD11b^−^CD169^+^CD68^+^CD80^lo^, or CD68^+^CD11b^−^, or F4/80^high^CD11b^low^, whereas the infiltrating bone marrow-derived monocytic macrophages are F4/80^+^CD11b^+^CD80^hi^ CD11b^+^ [23,34,35,40]. The data available for other mammalian species are limited. For humans, it has been proposed to use CD163L as a Kupffer cell-specific marker and CLEC5A as a marker specific for the monocyte-derived pro-inflammatory macrophages migrating to the liver [41].

**Figure 1:**
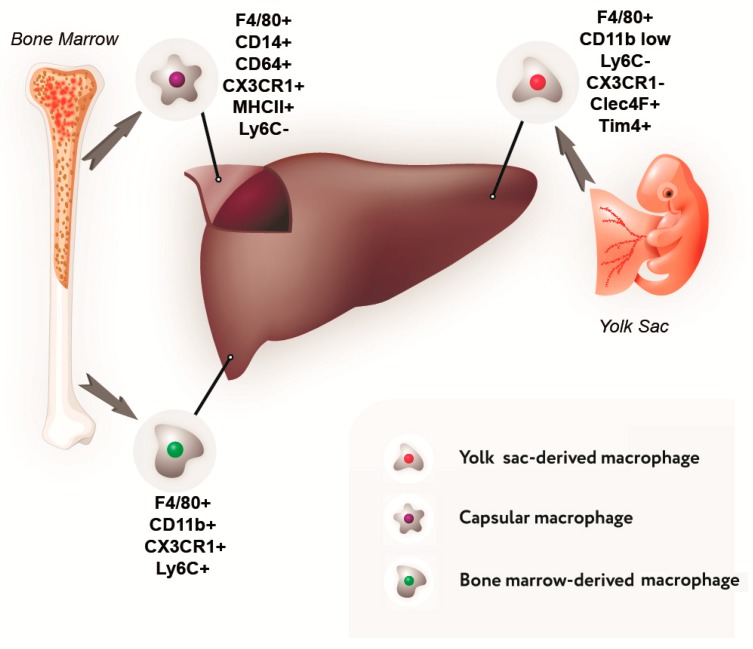
Sources of development and immunophenotype of a subpopulation of liver macrophages.

Data on the composition of the liver macrophage population and their phenotypic characteristics are summarized in Figure 1 and Table 1.

## 4. The Diversity of Macrophage Functional Types

The macrophages represent heterogeneous cell population not only by their developmental sources, but also by their functional characteristics. Macrophages are capable of very rapid phenotypic and functional changes under the action of signal molecules.

The main activators of Kupffer cells are complement factors C3a and C5a, as well as LPS [47,48]. LPS directly through TLR4 activates Kupffer cells, which leads to an increase in the synthesis and release of TNF-a, IL-1b, IL-6, IL-12, IL-18, IL-10, and IFN-γ. The binding of LPS and TLR4 leads to the formation of a multiprotein membrane complex containing myeloid differentiation factor MyD88, TNFR-associated factors (TRAFs), interleukin-1 receptor-associated kinases (IRAKs), and TGF-beta-activated kinase 1 (TAK1). The formation of this complex leads to the activation of JNK and p38 mitogen-activated protein kinases (MAPKs) and NF-kB signaling pathway were activated [5,47,48].

**Figure 2:**
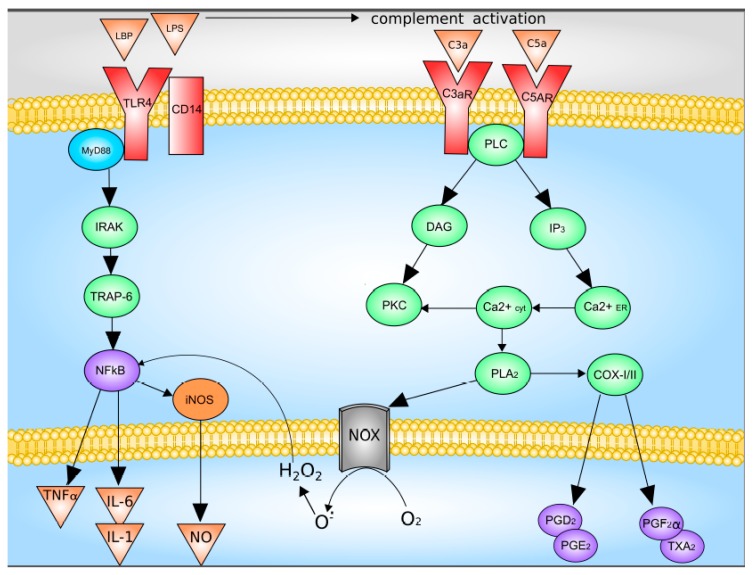
Mechanisms of Kupffer cell activation. C3 and C5, complement factor 3 and 5; C3a and C5a, activated complement factor 3 and 5; CD14, CD14 receptor; COX-I/II, cyclooxygenase-I/II; DAG, diacylglycerol; H_2_O_2_, hydrogen peroxide; iNOS, inducible nitric oxide synthase; IL-1, interleukin-1; IL-6, interleukin-6; IP3, inositol-3-phosphate; IRAK, interleukin-1 receptor-associated kinase; LPB, LPS-binding protein; LPS, lipopolysaccharide; NOX, NADPH oxidase; NFκB, nuclear factor κB; NO, nitric oxide; O_2_^−^, superoxide anion; PGD2, PGE2 and PGF2α, Prostaglandin D2, Prostaglandin E2, Prostaglandin F2α; PKC, protein kinase C; PLA2, phospholipase A2; PLC, phospholipase C; TLR4, Toll-like receptor 4; TNFα, tumor necrosis factor-α; TRAP-6, TNF-activated factor 6; TXA2, thromboxane A2. With modification made in NetworkPainter from [5] under CC-BY.

Upon activation of the complement system, C3a and C5a bind to their receptors with followed activation of down-stream signaling system which includes G proteins and phospholipase C. This pathway leads to increased synthesis of Kupffer cells Prostaglandin D2, Prostaglandin E2, Prostaglandin F2α, as well as thromboxane A2, as well as the release of superoxide [5,49]. Data on the Kupffer cells activation are summarized in Figure 2.

Another way to activate Kupffer cells is through the formation of inflammasomes—a complex of NOD-like receptors and apoptosis-associated speck-like protein a CARD, which is necessary for activation of caspase 1, which in turn activates pro-inflammatory cytokines IL-1β and IL-18 [50,51]. Formation of inflammasomes in the cytosol plays a central role in the development of inflammatory and fibrotic diseases. The most known for the development of liver diseases is the NLRP3 inflammasome, their increased formation leads to severe inflammation in the liver, which is associated with overproduction of IL-1β and severe liver neutrophilic infiltration [51].

In situ activation of macrophages (the so-called ‘macrophage polarization’) may take either the M1 pro-inflammatory direction or the M2 anti-inflammatory direction [47,52]. The M1 and M2 polar states of macrophage activation differ not only by specific markers, but, most importantly, by their roles in immune response and tissue repair [53]. A local shift towards M2 phenotype within a tissue damage area significantly improves the dynamics and efficiency of repair processes, as has been demonstrated for a number of particular models including skin wound healing [54], spinal cord damage [55], myocardial infarction and cardiomyopathy [56] and some other models. A shift in the balance of different functional classes of macrophages may occur by suppression of the M1 activation pathway as well as by reinforcement of the M2 activation pathway. At the same time, a part of macrophages may remain in the non-activated state [47,57]. In vivo shifts in the M1/M2 balance may be induced in a paracrine manner e.g., by blocking IL6 which promotes the M1 polarization [55] or introducing IL4 [58] or IL10 which promote the M2a and M2c polarization, respectively [59].

However, the use of markers is in many respects conditional. No exact correspondence exists between the Ly6C+ and M1 macrophages as well as between Ly6C- and M2 macrophages. On the contrary, Ly6C+ macrophages may in some cases give rise to both M1 and M2 functional types [60,61].

An experimental study by our research group has revealed that macrophages of bone-marrow origin and Kupffer cells have similar profiles of surface markers. Both of them express CD86 which is necessary for activation, proliferation, cytokine production and T cell differentiation [62], CD163 which is involved in bacterial recognition and triggering of local inflammatory reactions [63] and CD206 which is required for phagocytosis, antigen presentation and clearance of pro-inflammatory mediators from the circulation [64,65]. However, some quantitative differences in the expression of surface markers are evident: monocytic macrophages predominantly express the M1 marker CD86, whereas Kupffer cells predominantly express the M2 markers CD163 and CD206. At the same time, despite the association of surface markers with specific functionalities, the increased expression levels have been observed in both types of macrophages under the influence of bacterial endotoxin lipopolysaccharide (LPS) combined with IFN-γ or IL4 combined with IL10. Similar results have been obtained in the analysis of cytokine gene expression: transcription of pro-inflammatory cytokine genes (*Il1b*, *Il6*, *Tnfa*) and anti-inflammatory cytokine genes (*Il10*) in macrophages under the influence of LPS combined with IFN-γ or IL4 combined with IL10 becomes upregulated in concert [66].

To summarize, no strict border between M1 and M2 functional types of macrophages can be drawn. Instead, there is a continuous series of transitional forms being constantly updated to the needs of particular tissue microenvironments [48,67,68].

## 5. Macrophages and Regeneration of the Liver

The roles of macrophages in liver regeneration are diverse; first of all, it is the production of IL6 [69] and TNFα [70] which promote the transition of hepatocytes to mitotic cycle and also regulate hepatocyte apoptosis [5,71]. In addition, macrophages secrete the ligands of Wnt signaling pathway (also involved in the control of hepatocyte proliferation) [72] as well as TWEAK protein which activates proliferation of cholangiocytes and their transdifferentiation into hepatocytes [73].

It has been shown that both Kupffer cells and the transient immigrant macrophages of bone marrow (monocytic) origin can be involved in liver regeneration; their comparative involvement depends on the type of damage [25,27].

At the initial stages of the acute toxic injury induced by paracetamol or some other hepatotoxic substances, the numbers of the tissue-resident liver macrophages (Kupffer cells) decrease and the numbers of the blood monocyte-derived macrophages simultaneously increase, whereas the artificial suppression of the infiltration of the damaged liver with macrophages from the blood results in a more profound damage to the organ [74,75]. At later stages of the inflammatory process, the number of Kupffer cells within the liver is restored [25,27]. The artificial reduction in the proportion of Kupffer cells within the liver, especially at the early stages of regeneration or before the induction of the acute toxic injury, stimulates the proliferative response of hepatocytes [74,75]. The suppression of liver regeneration by Kupffer cells, in this case, is plausibly mediated by IL-18 released by Kupffer cells [27,76].

The sequence of participation of different macrophage populations in regeneration of the liver after the ischemia/reperfusion modeling is diametrically opposite: activation of Kupffer cells happens early while participation of the infiltrating monocyte-derived macrophages is delayed [77].

Yet another chain of events comes into action in the case of the compensatory growth following massive liver resections. The infiltration of hepatic tissue with monocytes and macrophages from the blood in this case is negligible [78] which correlates with low concentrations of MCP-1 in the remnant liver after 70% hepatectomy; the increased content of MCP-1 protein is responsible for chemotaxis of the bone marrow-derived (monocytic) macrophages after paracetamol- or tetrachloromethane-induced toxic injury [79]. Very few studies indicate the possibility of migration of the bone marrow-derived macrophages to the liver after partial hepatectomy, with the migration peaking on day 3 after the surgery [23,24]. As has been mentioned before, the discrepancies are probably related to the use of different markers.

Most likely, the lack of immigration of bone marrow-derived macrophages to the liver has no correlation with the resection volume. Our studies performed on rat model have demonstrated that subtotal liver resection causes a sharp decrease in the liver content of cytokine TNFa which is produced by Kupffer cells [44]. Under these conditions of low TNFa concentration, associated with the temporal growth arrest in hepatocytes characteristic of subtotal resection, immigration of an additional number of macrophages from the blood could rescue the TNFa levels, stimulate hepatocyte proliferation and thereby reduce the risks of fatal outcome for the entire organism. The potential success of such a strategy was confirmed by using a small size hepatic remnant model in mice under melatonin administration. The drug-promoted synthesis of TNFa and IL6 inside the remnant liver accelerated the proliferation of hepatocytes and stimulated the growth and functional recovery of the liver. The authors convincingly demonstrated that the beneficial increase in the hepatic levels of TNFa and IL6 was caused by infiltration of the remnant liver by monocytic macrophages from the blood [80].

Despite its potential usefulness, we observed no significant immigration of monocytic macrophages to the regenerating liver after subtotal liver resection in rats. At the same time, increased counts of CD68+ macrophages in the liver were observed on day 1 after the subtotal resection [44]. However, the CX3CR1^+^ and CD11b^hi^ cell counts were insignificant, which indicates negligible levels of the monocytic macrophage immigration to the liver at this point [44]. Probably, an increase in the number of liver macrophages after subtotal resection was provided solely by proliferation of Kupffer cells, the peak of which was observed at 48 h after surgery [44]. However, it cannot be excluded that there are genus- and species-specific features of the participation of monocytic macrophages in the post-hepatectomy liver regeneration. Another explanation for the apparent lack of the bone marrow-derived macrophages in the liver during the recovery after subtotal hepatectomy in rats may be related to the possible ‘damping’ of the expression of specific markers in monocytes upon their arrival in the remnant liver; such effects of molecular signature dissolution for particular cell populations have been described in mice and men under various conditions [81].

Despite the intense consideration given to the roles of macrophages in liver regeneration, it remains unclear why, after resections of different volume, the resident Kupffer cells participate in the reparative process on their own, without ‘a hand’ from the monocytes of the blood (especially considering that the bone marrow-derived macrophages largely influence the course of the recovery after the acute toxic liver injuries).

The reasons for the differential participation of various macrophage populations in the recovery of mammalian liver tissue can be considered from several perspectives. The suggestion about the diverse functions of the liver macrophages cannot be excluded. It has been shown that the CD11b+ macrophages are capable of robust production of cytokines but have low phagocytic activity, whereas CD68+ cells clearly indulge in phagocytosis [33]. The concomitant morphological differences are questionable; at least, in the liver, the number of smaller macrophages is about 8%, which roughly coincides with the estimated proportion of bone marrow-derived macrophages in this organ [15,25].

## 6. The Plasticity of Macrophages

High phenotypic plasticity is characteristic of macrophages regardless of their particular developmental sources of origin; yet some experts suggest that plasticity of macrophages may depend on their origin [60,61]. In recent years, the opinion has increasingly been expressed that, in mammals, macrophages of the bone marrow (monocytic) origin have the greatest plasticity. This concept is based on the diverse sensitivity of macrophages to activation factors; it is convincingly illustrated by differential gene expression in the monocytic and resident macrophages under the influence of LPS, as well as the increased sensitivity of resident macrophages to M-CSF [82,83,84]. In relation to the normal liver function and regeneration, this means that the bone marrow-derived monocytic macrophages are adapted to the needs and burdens of the liver under the inflammatory conditions while Kupffer cells, the tissue-resident liver macrophages, are adapted to the normal functioning of the liver [85]. An important point in the regulation of inflammatory reactions by monocytic macrophages is their ability to activate the neutrophil-mediated response e.g., production of free radicals and scavenging of apoptotic bodies [38].

A comparative study of the sensitivity of macrophages of different origin (exemplified by Kupffer cells and monocytic macrophages) to various activating factors was conducted in our laboratory. Both types of macrophages were shown to be more sensitive to the effects of LPS than to IL4 or IL10. However, monocytic macrophages were more sensitive to the effects of LPS as revealed by increased expression of interleukin genes *Il1b, Il6* and *Tnfa* under the influence of the lowest tested concentrations of LPS. In Kupffer cells, the low concentration of LPS noticeably upregulated the expression of the anti-inflammatory *Il10*, whereas the expression of genes for pro-inflammatory cytokines *Il1b, Il6* and *Tnfa* increased only under the influence of increased LPS concentrations [86]. The complementary analysis of gene expression profiles in monocytes revealed the increased expression of Toll-like receptor genes correlating with the increased sensitivity of the monocytes and monocytic macrophages to pro-inflammatory factors [87].

These results may indicate that Kupffer cells are predisposed (pre-differentiated) in some way towards the anti-inflammatory phenotype; yet a different explanation is plausible. A similar reaction to LPS is typical for the endotoxin tolerance, a constrained pro-inflammatory response which develops under the repeated action of bacterial endotoxin LPS and proceeds without the induction of the synthesis of pro-inflammatory cytokines (IL1b, IL6, and TNFa) but with an increase in production of anti-inflammatory cytokines (IL10) [88,89]. The reaction of Kupffer cells to the minimal concentrations of LPS in M1 differentiating medium, observed by us in this study, resembles the endotoxin tolerance by a number of features.

The LPS tolerance in Kupffer cells may develop as a consequence of continuous action of bacterial endotoxin that is formed inside the intestine, absorbed into the blood and primarily transferred to the liver [8]. It is not clear whether to regard this condition as the full-scale LPS tolerance, since according to our data it is accompanied by neither the increased expression of such characteristic LPS tolerance genes as *Socs1, Socs3, Irak1*, nor the reduced expression of *NF*-*kBp50* [90,91]. At the same time, expression of MAPK signaling-related genes *Erk2* and *p38* is reduced in the native Kupffer cells as compared with monocytic macrophages. The proteins ERK2 and P38 participate in the macrophage-mediated synthesis and secretion of pro-inflammatory cytokines; their decreased production may lead to LPS tolerance [89,90,91].

Additional data on differential phenotypic plasticity of macrophages of different origin were obtained on a mouse model of acetaminophen-induced acute liver injury. In this model, both the bone marrow-derived (monocytic) macrophages and the tissue-resident Kupffer cells are actively involved in the repair process. Some of the genes expressed at high levels in monocytic and resident macrophages turned out to be the same, for instance CD169 (*Siglec1*), F4/80 (*Emr1*), CD64 (*Fcgr1*), receptor tyrosine kinases Mer (*Mertk*) and Axl (*Axl*), macrosialin (*CD68*) and the MHC class II encoding genes. At the same time, the two macrophage subpopulations differentially expressed about 600 genes e.g., matrix metalloproteinase genes and the genes responsible for scavenging of dying cells [25]. It has been shown that macrophages of monocytic origin migrating to the liver initially express the genes of pro-inflammatory cytokines, but, under the influence of the microenvironment, their expression profile changes towards the synthesis of anti-inflammatory hepatoprotective cytokines; this finding highlights the high plasticity of bone marrow-derived macrophages [25]. In the course of regeneration, the resident liver macrophages (Kupffer cells) and the transient macrophages of monocytic origin produce a wide variety of pro-angiogenic factors; however, it is not clear which of the two subpopulations is the leading performer of this paracrine function [25,27].

Specialized participation of monocytic and resident macrophages in the regulation of homeostasis under different conditions might be considered from the prospects of coexistence of two macrophage lineages in mammals within the M1/M2 paradigm of functional specialization. It should be noted that the model, which originally implied domination of the two fully differentiated polar phenotypes, has been significantly updated [53,57,67]. The M1 and M2 phenotypes are currently viewed as the two extreme points in the continuous spectrum of functional types of macrophages. With this update, the search for specific quantitative markers for M1 and M2 phenotypes has become even more relevant and is constantly underway [47,48].

The analysis of our own data has shown that, although monocytes and Kupffer cells have different expression profiles, the differences are apparently unrelated to the M1/M2 paradigm. According to current definitions, the M1 phenotype is specifically marked by high expression of *Stat1 and* the iNOS gene *Nos2*, whereas the M2 phenotype is marked by expression of chemokines *Ccl17, Ccl24* and *Retnla* as well as high expression levels of *Arg1, Stat3* and *Stat6* [47,67].

It should nevertheless be noted that increased levels of expression observed for certain genes in Kupffer cells reflect their involvement in the maintenance of liver homeostasis, for example, in the hepatic blood flow regulation (*Nos2, Flt1, Kng1*) or principal biochemical cascades characteristic of the liver (*Arg1, C2, C6, C9, Crp*, and *Retnla*). High levels of expression of some surface markers, including CD163 and CD206 involved, respectively, in hemoglobin turnover and hormone metabolism, highlight the contribution of Kupffer cells to general functions of the liver as an organ [92,93].

Phagocytic activity and antigen presentation are the key functions of macrophages. The differences correlating with these functions may underlie the peculiarity of distribution of macrophage types in mammals. It has been shown that only the macrophages of bone marrow (monocytic) origin are present in dermis of the skin and loose fibrous connective tissue of the intestinal wall, that is, in the areas of utmost antigenic pressure. It has been found that bone marrow-derived macrophages that arrive in the liver following the ionizing irradiation treatment show more pronounced phagocytic activity as compared with the tissue-resident liver macrophages – Kupffer cells [82]. According to our own in vitro observations, within 1 h after the addition of latex particles to the medium, non-activated macrophages of monocytic origin show more pronounced phagocytic activity in comparison with Kupffer cells. At the same time, non-activated monocytic macrophages exhibit a constant level of endocytosis, while in Kupffer cells a sharp burst of phagocytic activity detected at the initial stages of the experiment is followed by a rapid decline. It is interesting to note that the addition of M1 or M2 polarization factors to the culture medium upregulates phagocytic activity in both types of macrophages [66,94].

The notable plasticity of bone marrow-derived macrophages has been demonstrated in experiments on replacement of the artificially depleted local resident macrophage populations with the monocytic macrophages arriving from the blood; the scheme works successfully for different organs including the liver [82,95,96].

## 7. The Niches of Macrophages

A number of authors adhere to an alternative interpretation of the ability of macrophages of bone marrow origin to replace resident macrophages. These researchers proceed from the assumption that the leading role in determining the properties of organ macrophages is played not by their source of origin, but by tight regulations conferred by local microenvironments [85,97]. The concept of ‘niche’ takes into account a set of factors under which a macrophage implements its potential, including the tissue topography, the state of the extracellular matrix, the preexisting diversity of macrophages and the interaction with other cell types through contacts or through the action of paracrine factors [83].

Each existing niche is described by several indicators: accessibility, vacancy and whether there is competition for a niche [83,85,97]. The ratio of these indicators gives the answer to a number of questions; for example, why only one macrophage population is present in the central nervous system (niches are not available for bone marrow-derived macrophages due to the hemato-encephalic barrier) [83,85,97]. In the liver, according to the authors of the concept, despite the availability of niches during the entire postnatal period, by the time active hematopoiesis is established in the red bone marrow, all niches are already occupied. Under the conditions of toxic injury, some niches become vacant, which causes migration of bone marrow-derived macrophages to the liver. By contrast, in the lungs, due to the specific nature of their operation, free niches appear constantly, but there is competition for them, which leads to a gradual increase in the proportion of bone marrow-derived pulmonary macrophages during the postnatal period [83,85,97].

Despite the fact that the macrophage niche concept is witty and useful from the prospects of functional studies, it still does not explain some of the most important phenomena associated with the growth and regeneration of the liver. First of all, this hypothesis postulates a gradual increase in the proportion of bone marrow-derived macrophages as the liver grows. The liver of laboratory rodents grows throughout life, while the proportion of bone marrow-derived macrophages in the liver of rats and mice remains approximately the same. After birth, the proportion of bone marrow-derived macrophages in the liver of mice reaches about 2-5% and remains at this level [16,17].

For a mouse model of toxic liver injury, it has been demonstrated that the immigrating bone marrow-derived macrophages do not survive and are replaced with proliferating tissue-resident macrophages [27]. Even less explainable in terms of the niche concept is the reaction of the macrophage system of the liver to resections. In accordance with the hypothesis [85,97], the growth of the organ is accompanied by the emergence of new macrophage niches, which should enhance macrophage immigration. However, the post-hepatectomy immigration of bone marrow macrophages is very limited or totally absent probably in connection with the low level of MCP-1 production in the liver remnant [78,79].

Thus, the macrophage niche hypothesis does not fully explain all the phenomena associated with the distribution of different types of macrophages in mammals, as well as their diverse roles in repair processes. It is likely that the source of origin still has a significant impact on the properties of macrophages including the proliferation capacity [98]. It has been shown that large and small liver macrophages have different proliferation capacities; the large cells, presumably of embryonic origin, are more capable of proliferation [28,29].

According to classical knowledge, the tissue-resident liver macrophages reside in the perisinusoidal space (of Disse). It turns out that this space accommodates several subpopulations of macrophages, differing in their properties and cell size [29,33]. In addition to these subpopulations, in the liver of mice, a subpopulation of macrophages of the organ capsule is described, which, unlike Kupffer cells, are of bone marrow origin; their main function is to protect the liver from pathogens that have entered the abdominal cavity. The number of such macrophages increases dramatically after the weaning, whereas the removal of capsular macrophages leads to a sharp increase in the number of neutrophils in the liver [45]. The role of capsular macrophages in liver regeneration remains obscure.

## 8. Macrophages in Liver Diseases

### 8.1. Alcoholic Liver Disease

Alcohol addiction affects millions of people around the world. Excessive alcohol consumption could lead to the development of alcoholic liver disease, fibrosis/cirrhosis, and hepatocellular carcinoma. The number of macrophages in the liver of patients with alcoholic liver disease and alcoholic hepatitis is significantly increased [99]. In patients with steatohepatitis with alcoholic disease, macrophages that were found in the liver expressed markers of both M1 and M2 macrophages [100]. In the blood of such patients, the concentration of cytokines (IL-6, IL-8, IL-18) and chemokines (monocyte chemoattractant protein 1 and macrophage inflammatory protein 1 alpha) increases, which also indicates macrophage activation in alcoholic liver disease [101]. Monocytes of patients with alcoholic liver disease are capable of spontaneous production of TNFa and are more sensitive to the effects of LPS [102]; a similar reaction is also observed in Kupffer cells of experimental animals with a model of alcoholic liver disease [103]. Monocytic infiltration (Ly-6C^+^ and Ly-6C^low^) in the liver is also observed in model animals; during phagocytosis of apoptosis dying hepatocytes, Ly-6C^+^ turned into Ly- Ly-6C^low^ [104]. Despite the key role of macrophages in the development of alcoholic liver disease, drugs that affect Kupffer’s cells in this pathology have not yet been used in the clinic. The use of antibodies to TNFa in patients with severe alcoholic illness did not significantly improve their clinical state [105].

### 8.2. Viral Hepatitis

Infection with hepatitis B and C viruses leads to the development of fibrosis/cirrhosis of the liver, and also increases the risk of hepatocytic carcinoma [1,2]. It is assumed that in the early stages of a viral infection, liver macrophages exhibit antiviral activity, but in chronic infections, liver macrophages could inhibit antiviral activity. So at the initial stages of a viral infection, Kupffer cells produce a large number of pro-inflammatory cytokines (IL-1β, IL-6, IL-18, and TNFα), while in chronic viral infections, macrophages begin to synthesize IL-10, TGFβ, galectin-9, PD-L1, and PD-L2, which suppresses antiviral defense [106]. The increase in the number of Kupffer cells in the liver expressing CD163, CD33, CD80, CD40, and MHC class-II is observed at hepatitis C virus infection [107,108]. At the same time, it is difficult to clearly distinguish what is the origin of these cells: monocytic or resident macrophages of the liver.

### 8.3. Liver Fibrosis

Hepatic stellate cells (HSCs), or Ito cells, are stromal cells and, according to various estimations, make up 5–15% of all liver cells [109]; they play a leading role in the development and liver fibrosis [110,111]. Activation of stellate liver cells can be induced by various factors. It has been shown that damaged endothelium cells begin to produce fibronectin, and are involved in the activation of TGFb1 [112]. Another source of growth factors are platelets that produce TGFb, PDGF, EGF [113]. Kupffer cells in response to liver damage secrete a large set of cytokines, primarily TGFb1, as well as lipid peroxidation products together with hepatocytes, which also activates HSCs [5,114]. At the next stage, active proliferation of HSCs is observed and PDGF is considered the main mitogen for them [115]. Under the influence PDGF, as well as MCP-1, HSCs migrate to chemokine sources [116] and begin to synthesize a large amount of collagen I [111]. In addition to stimulating the intercellular matrix synthesis the Kupffer cell also play a role in its remodeling due to the production of matrix metalloproteinases [8].

Due to the key role of HSCs in the development of liver fibrosis, these cells are considered as one of the main targets of therapeutic approaches [110,111]. To reduce liver fibrosis, it is proposed to direct the liver HSCs to a resting state by reducing the influence of profibrogenic factors [117,118] or by making them differentiate into hepatocyte-like cells [119]. Kupffer cells also could serve as possible targets of therapeutic approaches. In experimental liver fibrosis it has been shown that large number of monocytes migrate to the organ, and preventing such migration reduces the ability of the liver to degrade excess matrix [120]. Exposure the CCL2 inhibitor to the liver leads to an increase of Ly-6C^low^ number and accelerates the resolution of liver fibrosis [121].

### 8.4. Hepatocellular Carcinoma

Hepatocellular carcinoma is the most common liver tumor [122]. Tumor-associated macrophages play a leading role in tumor growth, angiogenesis, metastasis due to the production of a large number of growth factors: PDGFβ, VEGF, TGFβ, and EGFR ligands, cytokines: IL-6, TNFα, and IL-10, chemokines: CCL17, CCL22, CCL24, CXCL12, and IL-8, as well as other factors (MMPs, osteopontin, and cyclooxyganse-2) [122,123]. Tumor-associated macrophages are often identified by the immunophenotype CD68+ CD14+ and the number of macrophages correlates with tumor growth and prognosis [124]. Tumor-associated macrophages express glypican-3, and therefore, anti-glypican-3 antibodies are intended for therapeutic usage [125]. Another potential antitumor agent with tropism for macrophages is zoledronic acid [126].

## 9. Potential Therapeutic Approaches

Macrophages play a key in the regulation of liver homeostasis at normal and pathological conditions. In this regard, many authors consider macrophages as possible therapeutic targets [127,128,129] or specific agents, and change in the number of different macrophage populations could be considered as possible diagnostic or prognostic marker [9,130].

There are several experimental studies in which macrophages have been used as therapeutic agents for the treatment of liver fibrosis. Macrophages regulate the activity of Ito cells and synthesize a set of metalloproteinases involved in remodeling of the intercellular matrix [8]. Thus, in the mice model of liver fibrosis, it was found that after injection of bone marrow macrophages severity of fibrosis was decreased due to the activation of its own population of liver macrophages and the involvement of neutrophils, which actively secreted MMP9 and MMP13 [131]. In another study, injected macrophages from red bone marrow were preliminarily polarized to the M1 or M2 phenotype before administration [132]. Moreover, a positive effect was also detected after injection of M0, but transplantation of M1 macrophages led to a more pronounced reduction of fibrosis due to inhibition of activity of hepatic stellate cells. The transplantation of M2 macrophages had no effect [132]. The main limitation of the usage of macrophages as therapeutic agents is their pronounced phenotypic plasticity; therefore, the development of methods for obtaining macrophages with a stable phenotype is extremely critical.

In other works, macrophages are considered as therapeutic targets, acting on which in vivo could stimulate reparative processes in the liver [26]. Thus, in models of alcoholic and nonalcoholic liver disease, M2-polarized Kupffer cells induced apoptosis of Kupffer cells with an M1-phenotype and that had positive effect on the state of the liver [133]. Similar data were obtained on a model of acute pancreatitis in rats [134]. In pancreatitis, the accumulation of Kupffer cells with the M1 phenotype in the liver is observed. The induction of polarization of such macrophages toward M2 direction led to decreased inflammation in the pancreas [134].

A stimulating effect on reparative processes due to the effect on the macrophage population of the liver was also found in models of small-for-size organ syndrome [80,135]. It was found that transplantation of umbilical cord MSCs increases the proportion of M2 macrophages in the liver and stimulates the proliferation of macrophages in rats with removed more than 80% of the liver [128]. Administration of melatonin to rats with small-for-size syndrome causes liver macrophage infiltration, which leads to an increase of IL6 level and stimulation of hepatocyte proliferation [80].

In the case of inflammatory liver diseases, it is possible to block the migration of pro-inflammatory monocytes (Ly-6C+) into the liver, for example, by using CCL2 inhibitors [121] or to inhibit the activity of M-CSF, the level of which in the blood plasma of patients with hepatitis C is sharply increased, which leads to the accumulation of profibrotic macrophages in the liver [136]. Another way to reduce the activity of liver macrophages is to affect the intestinal flora. It has been shown that in patients with cirrhosis of the liver, a change in the intestinal flora is noted with contamination by more invasive bacteria [137]. The release of the intestinal flora from bacteria using broad-spectrum antibiotics and the colonization of the intestine with commensal bacteria can contribute to reparative processes in cirrhosis in the liver [138,139].

Another possible way is to use macrophages as prognostic or diagnostic markers in the clinic. This primarily relates to cancer. It has been established that the progression of tumor, including the liver cancer types, is closely related to the organ`s macrophage population, which provides angiogenesis, immunotolerance, etc. [140,141]. It was shown that macrophages of hepatic cell carcinoma carry a large number of markers of the so-called M2 phenotype, primarily CD163 and CD206 [142]. A particular prognostic interest is the presence of soluble sCD163, which is detected in blood plasma [143]. Detection of CD206+ cells in liver tumor tissue is also considered a poor prognostic marker [49,141]. However, the link of these markers with the progression of hepatic cell carcinoma needs further confirmation [141,143,144].

## 10. Summary and Conclusions

Macrophages are central players in regulation of tissue homeostasis; this is particularly well illustrated by the example of the liver. It is likely that the diversity of the functions of the liver itself has led to the formation of various subpopulations of macrophages adapted to participate in specific processes. The diversity of liver macrophages substantially complicates the investigation of their roles in liver regeneration. All liver macrophages respond to the organ damage, but their reactions are diverse and the comparative relevance of the changes to the reparative process is unclear. These complications may be resolved by exact assignments of specific molecular markers to different subpopulations of macrophages. Precise molecular portrayal of macrophage subpopulations may help to understand their particular roles in regeneration of the liver after injuries of different nature.

## Figures and Tables

**Table 1:** Liver macrophages subpopulations.

Species	Steady State	Liver Injury Model	Reference
Mouse	HemSCs: F4/80^+^ CD11b^hi^ EMPs: F4/80^bright^ CD11b^low^	-	[17]
Mouse	HemSCs: CD45+CD11b^hi^F4/80^hi^Ly6C^+^ EMPs: CD45^+^ CD11b^low^F4/80hiLy6C^−^	-	[18]
Rat	ED2 (CD163) ^high^ ED2 (CD163) ^dim^	-	[29]
Mouse	F4/80^+^Ly6C^+^CX3CR1^+^CD11b^hi^ F4/80^bright^Ly6C^−^CX3CR1^−^CD11b^low^	Acetaminophen-Induced Liver Injury F4/80^+^Ly6C^+^CX3CR1^+^CD11b^hi^ F4/80^bright^Ly6C^−^CX3CR1^−^CD11b^low^	[25]
Mouse	CD45^+^F4/80^high^CD11b^low^ D45^+^F4/80^low^CD11b^hig^	Acetaminophen-Induced Liver Injury	[27]
Rat	Large: CD68^+^CD163^+^ Small: CD68^+^ CD163^−^	-	[28,29]
Mouse	F4/80^+^CD11b^+^ F4/80^+^CD68^+^	PH F4/80^+^CD11b^+^ F4/80^+^CD68^+^	[23]
Rat	ED2^+^ (CD163): Mature Kupffer cells ED1^+^: mature and immature Kupffer cells	-	[42]
Mouse	F4/80^+^CD68^+^CD11b^+^, F4/80^+^CD68^+^CD11b^−^, F4/80^+^CD68^−^CD11b^+^, F4/80^+^CD68^−^CD11b^−^, F4/80^+^CD68^+^, F4/80^+^CD32^+^, F4/80^+^CD11b^+^	-	[24,33]
Human	CLEC5A^+^, CD163L^+^	-	[41]
Mouse	Sessile Kupffer cells Bone marrow–derived Kupffer cells	Virus-associated Intrahepatic inflammation	[43]
Human Mouse	CD68^+^CD206^+^	Liver fibrosis Parenchymal: CD68^+^CD206^+^ Scar-associated: M1:CD68^+^ IRF-5C^+^ M2: CD68^+^TGM-2^+^CD206^+^	[11]
Rat	CD68^+^ CD206^+^ Ly6C^−^ CX3CR1^−^	SR: CD68^+^, CD206^+^ Ly6C^−^ CX3CR1^−^	[44]
Mouse	CD169	PH: Increased proportion of CD169^+^ cells	[40]
	Capsular macrophages F4/80^+^CD14^+^CD64^+^ CX3CR1^+^MHCII ^+^Ly6C^−^		[45]
Mouse	Em-KCs: F4/80^+^CD11b^int^Clec4F^+^Tim4^+^ Mo-KCs: F4/80^+^CD11b^hi^Clec4F^−^ Tim4^−^		[46]
Human	Immunoregulatory KCs: CD68^+^MARCO^+^ Pro-inflammatory KCs: CD68^+^MARCO^−^	-	[36]
Mouse		Steatohepatitis KCs: Clec4f^+^ Mo-KCs: Lyz2^+^	[37]

Abbreviations. Hematopoietic stem cells, HemSCs; Erythro-myeloid progenitors, EMPs; Monocyte-derived Kupffer Cells, Mo-KCs; Embryonic Kupffer Cells, Em-KCs, Partial Hepatectomy, PH; Subtotal Resection, SR.

## Abbreviations

HGF	hepatocyte growth factor;
IL1	interleukin 1;
IL10	interleukin 10;
IL6	interleukin 6;
TGFb1	transforming growth factor β1;
TNFα	tumor necrosis factor α;
MCP-1	monocyte chemoattractant protein 1
PDGFβ	Platelet-derived growth factor subunit B
VEGF	Vascular endothelial growth factor
M-CSF	macrophage colony-stimulating factor
MMP-9	Matrix metallopeptidase 9
MMP-13	Matrix metallopeptidase 13
HemSCs	Hematopoietic stem cells
HSCs	Hepatic stellate cells
C3 and C5	complement factor 3 and 5;
C3a and C5a	activated complement factor 3 and 5;
CD14	CD14 receptor;
COX-I/II	cyclooxygenase-I/II;
DAG	diacylglycerol;
H_2_O_2_	hydrogen peroxide;
iNOS	inducible nitric oxide synthase;
IP3	inositol-3-phosphate;
IRAK	interleukin-1 receptor-associated kinase;
LPB	LPS-binding protein;
LPS	lipopolysaccharide;
NOX	NADPH oxidase;
NFκB	nuclear factor κB;
NO	nitric oxide;
O_2_^−^	superoxide anion;
PGD2, PGE2 and PGF2α,	Prostaglandin D2, Prostaglandin E2, Prostaglandin F2α;
PKC	protein kinase C;
PLA2	phospholipase A2;
PLC	phospholipase C;
TLR4	Toll-like receptor 4;
TRAP-6	TNF-activated factor 6;
TXA2	thromboxane A2
EMPs	Erythro-myeloid progenitors
Mo-KCs	Monocyte-derived Kupffer Cells
Em-KCs	Embryonic Kupffer Cells
MoMFs	Monocyte-derived macrophages
PH	Partial Hepatectomy
SR	Subtotal Resection

## References

[1] Tsochatzis E.A., Bosch J., Burroughs A.K. (2014). Liver cirrhosis. Lancet.

[2] Ganne-Carrié N. (2017). Epidemiology of liver cirrhosis. Rev. Prat..

[3] Nicolas C.T., Hickey R.D., Chen H.S., Mao S.A., Lopera Higuita M., Wang Y., Nyberg S.L. (2017). Concise Review: Liver Regenerative Medicine: From Hepatocyte Transplantation to Bioartificial Livers and Bioengineered Grafts. Stem Cells.

[4] Zhang J., Zhao X., Liang L., Li J., Demirci U., Wang S. (2018). A decade of progress in liver regenerative medicine. Biomaterials.

[5] Bilzer M., Roggel F., Gerbes A.L. (2006). Role of Kupffer cells in host defense and liver disease. Liver Int..

[6] Liaskou E., Wilson D.V., Oo Y.H. (2012). Innate immune cells in liver inflammation. Mediators Inflamm..

[7] Dong X., Liu J., Xu Y., Cao H. (2019). Role of macrophages in experimental liver injury and repair in mice. Exp. Ther. Med..

[8] Michalopoulos G.K. (2014). Advances in liver regeneration. Expert Rev. Gastroenterol. Hepatol..

[9] Guillot A., Tacke F. (2019). Liver Macrophages: Old Dogmas and New Insights. Hepatol. Commun..

[10] Wynn T.A., Barron L. (2010). Macrophages: master regulators of inflammation and fibrosis. Semin. Liver Dis..

[11] Beljaars L., Schippers M., Reker-Smit C., Martinez F.O., Helming L., Poelstra K., Melgert B.N. (2014). Hepatic Localization of Macrophage Phenotypes during Fibrogenesis and Resolution of Fibrosis in Mice and Humans. Front. Immunol..

[12] Tacke F. (2017). Targeting hepatic macrophages to treat liver diseases. J. Hepatol..

[13] Keirsse J., Van Damme H., Geeraerts X., Beschin A., Raes G., Van Ginderachter J.A. (2018). The role of hepatic macrophages in liver metastasis. Cell. Immunol..

[14] Yeh C.-C., Chao K.-C., Huang S.J. (2013). Innate Immunity, Decidual Cells, and Preeclampsia. Reprod. Sci..

[15] Chazaud B. (2014). Macrophages: Supportive cells for tissue repair and regeneration. Immunobiology.

[16] Gomez Perdiguero E., Klapproth K., Schulz C., Busch K., Azzoni E., Crozet L., Garner H., Trouillet C., De Bruijn M.F., Geissmann F. (2015). Tissue-resident macrophages originate from yolk-sac-derived erythro-myeloid progenitors. Nature.

[17] Perdiguero E.G., Geissmann F. (2016). The development and maintenance of resident macrophages. Nat. Immunol..

[18] Hoeffel G., Chen J., Lavin Y., Low D., Almeida F.F., See P., Beaudin A.E., Lum J., Low I., Forsberg E.C. (2015). C-Myb(+) erythro-myeloid progenitor-derived fetal monocytes give rise to adult tissue-resident macrophages. Immunity.

[19] Hoeffel G., Ginhoux F. (2018). Fetal monocytes and the origins of tissue-resident macrophages. Cell. Immunol..

[20] Goldmann T., Wieghofer P., Jordão M.J.C., Prutek F., Hagemeyer N., Frenzel K., Amann L., Staszewski O., Kierdorf K., Krueger M. (2016). Origin, fate and dynamics of macrophages at central nervous system interfaces. Nat. Immunol..

[21] Kopf M., Schneider C., Nobs S.P. (2015). The development and function of lung-resident macrophages and dendritic cells. Nat. Immunol..

[22] Epelman S., Lavine K.J., Randolph G.J. (2014). Origin and Functions of Tissue Macrophages. Immunity.

[23] Nishiyama K., Nakashima H., Ikarashi M., Kinoshita M., Nakashima M., Aosasa S., Seki S., Yamamoto J. (2015). Mouse CD11b+Kupffer cells recruited from bone marrow accelerate liver regeneration after partial hepatectomy. PLoS ONE.

[24] Ikarashi M., Nakashima H., Kinoshita M., Sato A., Nakashima M., Miyazaki H., Nishiyama K., Yamamoto J., Seki S. (2013). Distinct development and functions of resident and recruited liver Kupffer cells/macrophages. J. Leukoc. Biol..

[25] Zigmond E., Samia-Grinberg S., Pasmanik-Chor M., Brazowski E., Shibolet O., Halpern Z., Varol C. (2014). Infiltrating Monocyte-Derived Macrophages and Resident Kupffer Cells Display Different Ontogeny and Functions in Acute Liver Injury. J. Immunol..

[26] Ju C., Tacke F. (2016). Hepatic macrophages in homeostasis and liver diseases: from pathogenesis to novel therapeutic strategies. Cell. Mol. Immunol..

[27] You Q., Holt M., Yin H., Li G., Hu C.J., Ju C. (2013). Role of hepatic resident and infiltrating macrophages in liver repair after acute injury. Biochem. Pharmacol..

[28] Armbrust T., Ramadori G. (1996). Functional characterization of two different Kupffer cell populations of normal rat liver. J. Hepatol..

[29] He Y., Sadahiro T., Noh S.I., Wang H., Todo T., Chai N., Klein A.S., Wu G.D. (2009). Flow cytometric isolation and phenotypic characterization of two subsets of ED2+ (CD163) hepatic macrophages in rats. Hepatol. Res..

[30] Gottfried E., Kunz-Schughart L.A., Weber A., Rehli M., Peuker A., Müller A., Kastenberger M., Brockhoff G., Andreesen R., Kreutz M. (2008). Expression of CD68 in Non-Myeloid Cell Types. Scand. J. Immunol..

[31] Schittenhelm L., Hilkens C.M., Morrison V.L. (2017). β2 Integrins As Regulators of Dendritic Cell, Monocyte, and Macrophage Function. Front. Immunol..

[32] Sanchez-Madrid F., Simon P., Thompson S., Springer T.A. (1983). Mapping of antigenic and functional epitopes on the alpha- and beta-subunits of two related mouse glycoproteins involved in cell interactions, LFA-1 and Mac-1. J. Exp. Med..

[33] Kinoshita M., Uchida T., Sato A., Nakashima M., Nakashima H., Shono S., Habu Y., Miyazaki H., Hiroi S., Seki S. (2010). Characterization of two F4/80-positive Kupffer cell subsets by their function and phenotype in mice. J. Hepatol..

[34] Movita D., Kreefft K., Biesta P., van Oudenaren A., Leenen P.J.M., Janssen H.L.A., Boonstra A. (2012). Kupffer cells express a unique combination of phenotypic and functional characteristics compared with splenic and peritoneal macrophages. J. Leukoc. Biol..

[35] Haldar M., Murphy K.M. (2014). Origin, development, and homeostasis of tissue-resident macrophages. Immunol. Rev..

[36] MacParland S.A., Liu J.C., Ma X.Z., Innes B.T., Bartczak A.M., Gage B.K., Manuel J., Khuu N., Echeverri J., Linares I. (2018). Single cell RNA sequencing of human liver reveals distinct intrahepatic macrophage populations. Nat. Commun..

[37] Krenkel O., Hundertmark J., Abdallah A.T., Kohlhepp M., Puengel T., Roth T., Branco D.P.P., Mossanen J.C., Luedde T., Trautwein C. (2019). Myeloid cells in liver and bone marrow acquire a functionally distinct inflammatory phenotype during obesity-related steatohepatitis. Gut.

[38] Graubardt N., Vugman M., Mouhadeb O., Caliari G., Pasmanik-Chor M., Reuveni D., Zigmond E., Brazowski E., David E., Chappell-Maor L. (2017). Ly6Chimonocytes and their macrophage descendants regulate neutrophil function and clearance in acetaminophen-induced liver injury. Front. Immunol..

[39] Yin S., Wang H., Park O., Wei W., Shen J., Gao B. (2011). Enhanced liver regeneration in IL-10-deficient mice after partial hepatectomy via stimulating inflammatory response and activating hepatocyte STAT3. Am. J. Pathol..

[40] Behnke K., Zhuang Y., Xu H.C., Sundaram B., Reich M., Shinde P.V., Huang J., Modares N.F., Tumanov A.V., Polz R. (2018). B Cell-Mediated Maintenance of Cluster of Differentiation 169-Positive Cells Is Critical for Liver Regeneration. Hepatology.

[41] González-Domínguez É., Samaniego R., Flores-Sevilla J.L., Campos-Campos S.F., Gómez-Campos G., Salas A., Campos-Peña V., Corbí Á.L., Sánchez-Mateos P., Sánchez-Torres C. (2015). CD163L1 and CLEC5A discriminate subsets of human resident and inflammatory macrophages in vivo. J. Leukoc. Biol..

[42] Xiang S., Dong H.H., Liang H.F., He S.Q., Zhang W., Li C.H., Zhang B.X., Zhang B.H., Jing K., Tomlinson S. (2012). Oval cell response is attenuated by depletion of liver resident macrophages in the 2-AAF/partial hepatectomy rat. PLoS ONE.

[43] Klein I., Cornejo J.C., Polakos N.K., John B., Wuensch S.A., Topham D.J., Pierce R.H., Crispe I.N. (2007). Kupffer cell heterogeneity: Functional properties of bone marrow-derived and sessile hepatic macrophages. Blood.

[44] Elchaninov A.V., Fatkhudinov T.K., Usman N.Y., Kananykhina E.Y., Arutyunyan I.V., Makarov A.V., Lokhonina A.V., Eremina I.Z., Surovtsev V.V., Goldshtein D.V. (2018). Dynamics of macrophage populations of the liver after subtotal hepatectomy in rats. BMC Immunol..

[45] Sierro F., Evrard M., Rizzetto S., Melino M., Mitchell A.J., Florido M., Beattie L., Walters S.B., Tay S.S., Lu B. (2017). A Liver Capsular Network of Monocyte-Derived Macrophages Restricts Hepatic Dissemination of Intraperitoneal Bacteria by Neutrophil Recruitment. Immunity.

[46] Scott C.L., Zheng F., De Baetselier P., Martens L., Saeys Y., De Prijck S., Lippens S., Abels C., Schoonooghe S., Raes G. (2016). Bone marrow-derived monocytes give rise to self-renewing and fully differentiated Kupffer cells. Nat. Commun..

[47] Murray P.J., Allen J.E., Biswas S.K., Fisher E.A., Gilroy D.W., Goerdt S., Gordon S., Hamilton J.A., Ivashkiv L.B., Lawrence T. (2014). Macrophage Activation and Polarization: Nomenclature and Experimental Guidelines. Immunity.

[48] Malyshev I., Malyshev Y. (2015). Current Concept and Update of the Macrophage Plasticity Concept: Intracellular Mechanisms of Reprogramming and M3 Macrophage “Switch” Phenotype. Biomed Res. Int..

[49] Ren C.-X., Leng R.-X., Fan Y.-G., Pan H.-F., Li B.-Z., Wu C.-H., Wu Q., Wang N.-N., Xiong Q.-R., Geng X.-P. (2017). Intratumoral and peritumoral expression of CD68 and CD206 in hepatocellular carcinoma and their prognostic value. Oncol. Rep..

[50] Mehal W.Z. (2014). The inflammasome in liver injury and non-alcoholic fatty liver disease. Dig. Dis..

[51] Wree A., Eguchi A., Mcgeough M.D., Pena C.A., Johnson C.D., Canbay A., Hoffman H.M., Feldstein A.E. (2014). NLRP3 inflammasome activation results in hepatocyte pyroptosis, liver inflammation, and fibrosis in mice. Hepatology.

[52] Murray P.J., Wynn T.A. (2011). Obstacles and opportunities for understanding macrophage polarization. J. Leukoc. Biol..

[53] Martinez F.O., Gordon S. (2014). The M1 and M2 paradigm of macrophage activation: time for reassessment. F1000Prime Rep..

[54] Okizaki S., Ito Y., Hosono K., Oba K., Ohkubo H., Amano H., Shichiri M., Majima M. (2015). Suppressed recruitment of alternatively activated macrophages reduces TGF-β1 and impairs wound healing in streptozotocin-induced diabetic mice. Biomed. Pharmacother..

[55] Nakajima H., Uchida K., Guerrero A.R., Watanabe S., Sugita D., Takeura N., Yoshida A., Long G., Wright K.T., Johnson W.E.B. (2012). Transplantation of Mesenchymal Stem Cells Promotes an Alternative Pathway of Macrophage Activation and Functional Recovery after Spinal Cord Injury. J. Neurotrauma.

[56] Singla D.K., Singla R.D., Abdelli L.S., Glass C. (2015). Fibroblast Growth Factor-9 Enhances M2 Macrophage Differentiation and Attenuates Adverse Cardiac Remodeling in the Infarcted Diabetic Heart. PLoS ONE.

[57] Murray P.J. (2017). Macrophage Polarization. Annu. Rev. Physiol..

[58] Kiguchi N., Kobayashi Y., Saika F., Sakaguchi H., Maeda T., Kishioka S. (2015). Peripheral interleukin-4 ameliorates inflammatory macrophage-dependent neuropathic pain. Pain.

[59] Yang S.-L., Chen S.-L., Wu J.-Y., Ho T.-C., Tsao Y.-P. (2010). Pigment epithelium-derived factor induces interleukin-10 expression in human macrophages by induction of PPAR gamma. Life Sci..

[60] Kole A., Kelsall B.L., Valatas V., He J., Rivollier A. (2012). Inflammation switches the differentiation program of Ly6C hi monocytes from antiinflammatory macrophages to inflammatory dendritic cells in the colon. J. Exp. Med..

[61] Bain C.C., Scott C.L., Uronen-Hansson H., Gudjonsson S., Jansson O., Grip O., Guilliams M., Malissen B., Agace W.W., Mowat A.M. (2013). Resident and pro-inflammatory macrophages in the colon represent alternative context-dependent fates of the same Ly6Chi monocyte precursors. Mucosal Immunol..

[62] Yin G., Zhu X., Guo C., Yang Y., Han T., Chen L., Yin W., Gao P., Zhang H., Geng J. (2013). Differential expression of estradiol and estrogen receptor α in severe preeclamptic pregnancies compared with normal pregnancies. Mol. Med. Rep..

[63] Fabriek B.O., van Bruggen R., Deng D.M., Ligtenberg A.J.M., Nazmi K., Schornagel K., Vloet R.P.M., Dijkstra C.D., van den Berg T.K. (2009). The macrophage scavenger receptor CD163 functions as an innate immune sensor for bacteria. Blood.

[64] Martinez-Pomares L. (2012). The mannose receptor. J. Leukoc. Biol..

[65] Stahl P.D., Ezekowitz R.A. (1998). The mannose receptor is a pattern recognition receptor involved in host defense. Curr. Opin. Immunol..

[66] Lokhonina A., Elchaninov A., Fatkhudinov T., Makarov A., Arutyunyan I., Grinberg M., Glinkina V., Surovtsev V., Bolshakova G., Goldshtein D. (2019). Activated Macrophages of Monocytic Origin Predominantly Express Proinflammatory Cytokine Genes, Whereas Kupffer Cells Predominantly Express Anti-Inflammatory Cytokine Genes. Biomed Res. Int..

[67] Orecchioni M., Ghosheh Y., Pramod A.B., Ley K. (2019). Macrophage Polarization: Different Gene Signatures in M1(LPS+) vs. Classically and M2(LPS–) vs. Alternatively Activated Macrophages. Front. Immunol..

[68] Jablonski K.A., Amici S.A., Webb L.M., de Dios Ruiz-Rosado J., Popovich P.G., Partida-Sanchez S., Guerau-de-Arellano M. (2015). Novel Markers to Delineate Murine M1 and M2 Macrophages. PLoS ONE.

[69] Cressman D.E., Greenbaum L.E., DeAngelis R.A., Ciliberto G., Furth E.E., Poli V., Taub R. (1996). Liver failure and defective hepatocyte regeneration in interleukin-6- deficient mice. Science..

[70] Webber E.M., Bruix J., Pierce R.H., Fausto N. (1998). Tumor necrosis factor primes hepatocytes for DNA replication in the rat. Hepatology.

[71] Michalopoulos G.K. (2010). Liver regeneration after partial hepatectomy: Critical analysis of mechanistic dilemmas. Am. J. Pathol..

[72] Yang J., Mowry L.E., Nejak-Bowen K.N., Okabe H., Diegel C.R., Lang R.A., Williams B.O., Monga S.P. (2014). β-catenin signaling in murine liver zonation and regeneration: a Wnt-Wnt situation!. Hepatology.

[73] Bird T.G., Lu W.-Y., Boulter L., Gordon-Keylock S., Ridgway R.A., Williams M.J., Taube J., Thomas J.A., Wojtacha D., Gambardella A. (2013). Bone marrow injection stimulates hepatic ductular reactions in the absence of injury via macrophage-mediated TWEAK signaling. Proc. Natl. Acad. Sci. USA.

[74] Meijer C., Wiezer M.J., Diehl A.M., Schouten H.J., Schouten H.J., Meijer S., van Rooijen N., van Lambalgen A.A., Dijkstra C.D., van Leeuwen P.A. (2000). Kupffer cell depletion by CI2MDP-liposomes alters hepatic cytokine expression and delays liver regeneration after partial hepatectomy. Liver.

[75] Constandinou C.M., Vuthoori S., Iredale J.P., Partolina M., Lang R., Clay S., Duffield J.S., Wu S., Forbes S.J. (2008). Selective depletion of macrophages reveals distinct, opposing roles during liver injury and repair. J. Clin. Investig..

[76] Xu C.S., Jiang Y., Zhang L.X., Chang C.F., Wang G.P., Shi R.J., Yang Y.J. (2012). The role of kupffer cells in rat liver regeneration revealed by cell-specific microarray analysis. J. Cell. Biochem..

[77] Zhai Y., Busuttil R.W., Kupiec-Weglinski J.W. (2011). Liver ischemia and reperfusion injury: New insights into mechanisms of innate-adaptive immune-mediated tissue inflammation. Am. J. Transplant..

[78] Michalopoulos G.K. (2011). Liver regeneration: alternative epithelial pathways. Int. J. Biochem. Cell Biol..

[79] Wyler S.L., D’Ingillo S.L., Lamb C.L., Mitchell K.A. (2016). Monocyte chemoattractant protein-1 is not required for liver regeneration after partial hepatectomy. J. Inflamm..

[80] Song Z., Humar B., Gupta A., Maurizio E., Borgeaud N., Graf R., Clavien P.-A., Tian Y. (2018). Exogenous melatonin protects small-for-size liver grafts by promoting monocyte infiltration and releases interleukin-6. J. Pineal Res..

[81] Lampé R., Kövér Á., Szűcs S., Pál L., Árnyas E., Ádány R., Póka R. (2015). Phagocytic index of neutrophil granulocytes and monocytes in healthy and preeclamptic pregnancy. J. Reprod. Immunol..

[82] Beattie L., Sawtell A., Mann J., Frame T.C.M., Teal B., de Labastida Rivera F., Brown N., Walwyn-Brown K., Moore J.W.J., MacDonald S. (2016). Bone marrow-derived and resident liver macrophages display unique transcriptomic signatures but similar biological functions. J. Hepatol..

[83] T’Jonck W., Guilliams M., Bonnardel J. (2018). Niche signals and transcription factors involved in tissue-resident macrophage development. Cell. Immunol..

[84] Pridans C., Sauter K.A., Irvine K.M., Davis G.M., Lefevre L., Raper A., Rojo R., Nirmal A.J., Beard P., Cheeseman M. (2017). Macrophage colony-stimulating factor increases hepatic macrophage content, liver growth, and lipid accumulation in neonatal rats. Am. J. Physiol. Liver Physiol..

[85] Guilliams M., Scott C.L. (2017). Does niche competition determine the origin of tissue-resident macrophages?. Nat. Rev. Immunol..

[86] Lokhonina A.V., Elchaninov A.V., Makarov A.V., Nikitina M.P., Goldshtein D.V., Paltsev M.A., Fatkhudinov T.K. (2019). Comparative characteristics of the susceptibility of kupffer cells and macrophages of bone-background origin to activation factors. Mol. Meditsina.

[87] Nikitina M.P., Lokhonina A.V., Elchaninov A.V., Makarov A.V., Tagirova M.K., Grinberg M.V., Glinkina V.V., Bolshakova G.B., Goldshtein D.V., Fatkhudinov T.K. (2019). Comparative analysis of gene expression profiles in Kupffer cells and monocytes. Bull. Exp. Biol. Med..

[88] West M.A., Heagy W. (2002). Endotoxin tolerance: A review. Crit. Care Med..

[89] Liu D., Cao S., Zhou Y., Xiong Y. (2019). Recent advances in endotoxin tolerance. J. Cell. Biochem..

[90] Qin H., Roberts K.L., Niyongere S.A., Cong Y., Elson C.O., Benveniste E.N. (2007). Molecular Mechanism of Lipopolysaccharide-Induced SOCS-3 Gene Expression in Macrophages and Microglia. J. Immunol..

[91] Nimah M., Zhao B., Denenberg A.G., Bueno O., Molkentin J., Wong H.R., Shanley T.P. (2005). Contribution of MKP-1 regulation of p38 to endotoxin tolerance. Shock.

[92] Italiani P., Boraschi D. (2014). From Monocytes to M1/M2 Macrophages: Phenotypical vs. Functional Differentiation. Front. Immunol..

[93] Teh Y.C., Ding J.L., Ng L.G., Chong S.Z. (2019). Capturing the Fantastic Voyage of Monocytes Through Time and Space. Front. Immunol..

[94] Lokhonina A.V., Makarov A.V., Elchaninov A.V., Arutyunyan I.V., Shmakova T.V., Grinberg M.V., Usman N.Y., Surovtsev V.V., Chernikov V.P., Fatkhudinov T.K. (2019). Quantitative and Qualitative Characterization of Phagocytic Activity of Macrophages of Bone Marrow and Fetal Origin. Bull. Exp. Biol. Med..

[95] van de Laar L., Saelens W., De Prijck S., Martens L., Scott C.L., Van Isterdael G., Hoffmann E., Beyaert R., Saeys Y., Lambrecht B.N. (2016). Yolk Sac Macrophages, Fetal Liver, and Adult Monocytes Can Colonize an Empty Niche and Develop into Functional Tissue-Resident Macrophages. Immunity.

[96] Merlin S., Bhargava K.K., Ranaldo G., Zanolini D., Palestro C.J., Santambrogio L., Prat M., Follenzi A., Gupta S. (2016). Kupffer Cell Transplantation in Mice for Elucidating Monocyte/Macrophage Biology and for Potential in Cell or Gene Therapy. Am. J. Pathol..

[97] Bonnardel J., Guilliams M. (2018). Developmental control of macrophage function. Curr. Opin. Immunol..

[98] Röszer T. (2018). Understanding the Biology of Self-Renewing Macrophages. Cells.

[99] Karakucuk I., Dilly S.A., Maxwell J.D. (1989). Portal tract macrophages are increased in alcoholic liver disease. Histopathology.

[100] Lee J., French B., Morgan T., French S.W. (2014). The liver is populated by a broad spectrum of markers for macrophages. In alcoholic hepatitis the macrophages are M1 and M2. Exp. Mol. Pathol..

[101] Fisher N.C., Neil D.A., Williams A., Adams D.H. (1999). Serum concentrations and peripheral secretion of the beta chemokines monocyte chemoattractant protein 1 and macrophage inflammatory protein 1alpha in alcoholic liver disease. Gut.

[102] Gobejishvili L., Barve S., Joshi-Barve S., Uriarte S., Song Z., McClain C. (2006). Chronic ethanol-mediated decrease in cAMP primes macrophages to enhanced LPS-inducible NF-kappaB activity and TNF expression: relevance to alcoholic liver disease. Am. J. Physiol. Gastrointest. Liver Physiol..

[103] Mandrekar P., Ambade A., Lim A., Szabo G., Catalano D. (2011). An essential role for monocyte chemoattractant protein-1 in alcoholic liver injury: regulation of proinflammatory cytokines and hepatic steatosis in mice. Hepatology.

[104] Wang M., You Q., Lor K., Chen F., Gao B., Ju C. (2014). Chronic alcohol ingestion modulates hepatic macrophage populations and functions in mice. J. Leukoc. Biol..

[105] Naveau S., Chollet-Martin S., Dharancy S., Mathurin P., Jouet P., Piquet M.-A., Davion T., Oberti F., Broët P., Emilie D. (2004). A double-blind randomized controlled trial of infliximab associated with prednisolone in acute alcoholic hepatitis. Hepatology.

[106] Tu Z., Pierce R.H., Kurtis J., Kuroki Y., Crispe I.N., Orloff M.S. (2010). Hepatitis C virus core protein subverts the antiviral activities of human Kupffer cells. Gastroenterology.

[107] Burgio V.L., Ballardini G., Artini M., Caratozzolo M., Bianchi F.B., Levrero M. (1998). Expression of co-stimulatory molecules by Kupffer cells in chronic hepatitis of hepatitis C virus etiology. Hepatology.

[108] Dolganiuc A., Norkina O., Kodys K., Catalano D., Bakis G., Marshall C., Mandrekar P., Szabo G. (2007). Viral and host factors induce macrophage activation and loss of toll-like receptor tolerance in chronic HCV infection. Gastroenterology.

[109] Hellerbrand C. (2013). Hepatic stellate cells - The pericytes in the liver. Pflugers Arch. Eur. J. Physiol..

[110] Friedman S.L. (2008). Hepatic stellate cells: Protean, multifunctional, and enigmatic cells of the liver. Physiol. Rev..

[111] Higashi T., Friedman S.L., Hoshida Y. (2017). Hepatic stellate cells as key target in liver fibrosis. Adv. Drug Deliv. Rev..

[112] Jarnagin W.R., Rockey D.C., Koteliansky V.E., Wang S.S., Montgomery Bissell D. (1994). Expression of variant fibronectins in wound healing: Cellular source and biological activity of the EIIIA segment in rat hepatic fibrogenesis. J. Cell Biol..

[113] Bachem M.G., Melchior R., Gressner A.M. (1989). The role of thrombocytes in liver fibrogenesis: effects of platelet lysate and thrombocyte-derived growth factors on the mitogenic activity and glycosaminoglycan synthesis of cultured rat liver fat storing cells. J. Clin. Chem. Clin. Biochem..

[114] Novo E., Marra F., Zamara E., Valfrè Di Bonzo L., Caligiuri A., Cannito S., Antonaci C., Colombatto S., Pinzani M., Parola M. (2006). Dose dependent and divergent effects of superoxide anion on cell death, proliferation, and migration of activated human hepatic stellate cells. Gut.

[115] Pinzani M. (2002). PDGF and signal transduction in hepatic stellate cells. Front. Biosci..

[116] Kinnman N., Hultcrantz R., Barbu V., Rey C., Wendum D., Poupon R., Housset C. (2000). PDGF-mediated chemoattraction of hepatic stellate cells by bile duct segments in cholestatic liver injury. Lab. Invest..

[117] Kisseleva T., Cong M., Paik Y.H., Scholten D., Jiang C., Benner C., Iwaisako K., Moore-Morris T., Scott B., Tsukamoto H. (2012). Myofibroblasts revert to an inactive phenotype during regression of liver fibrosis. Proc. Natl. Acad. Sci. USA.

[118] Troeger J.S., Mederacke I., Gwak G.Y., Dapito D.H., Mu X., Hsu C.C., Pradere J.P., Friedman R.A., Schwabe R.F. (2012). Deactivation of hepatic stellate cells during liver fibrosis resolution in mice. Gastroenterology.

[119] Song G., Pacher M., Balakrishnan A., Yuan Q., Tsay H.C., Yang D., Reetz J., Brandes S., Dai Z., Pützer B.M. (2016). Direct Reprogramming of Hepatic Myofibroblasts into Hepatocytes in Vivo Attenuates Liver Fibrosis. Cell Stem Cell.

[120] Ramachandran P., Pellicoro A., Vernon M.A., Boulter L., Aucott R.L., Ali A., Hartland S.N., Snowdon V.K., Cappon A., Gordon-Walker T.T. (2012). Differential Ly-6C expression identifies the recruited macrophage phenotype, which orchestrates the regression of murine liver fibrosis. Proc. Natl. Acad. Sci. USA.

[121] Baeck C., Wehr A., Karlmark K.R., Heymann F., Vucur M., Gassler N., Huss S., Klussmann S., Eulberg D., Luedde T. (2012). Pharmacological inhibition of the chemokine CCL2 (MCP-1) diminishes liver macrophage infiltration and steatohepatitis in chronic hepatic injury. Gut.

[122] Capece D., Fischietti M., Verzella D., Gaggiano A., Cicciarelli G., Tessitore A., Zazzeroni F., Alesse E. (2013). The inflammatory microenvironment in hepatocellular carcinoma: a pivotal role for tumor-associated macrophages. Biomed Res. Int..

[123] Zhang Q., Lou Y., Bai X.-L., Liang T.-B. (2018). Immunometabolism: A novel perspective of liver cancer microenvironment and its influence on tumor progression. World J. Gastroenterol..

[124] Ding T., Xu J., Wang F., Shi M., Zhang Y., Li S.-P., Zheng L. (2009). High tumor-infiltrating macrophage density predicts poor prognosis in patients with primary hepatocellular carcinoma after resection. Hum. Pathol..

[125] Ikeda M., Ohkawa S., Okusaka T., Mitsunaga S., Kobayashi S., Morizane C., Suzuki I., Yamamoto S., Furuse J. (2014). Japanese phase I study of GC33, a humanized antibody against glypican-3 for advanced hepatocellular carcinoma. Cancer Sci..

[126] Rogers T.L., Wind N., Hughes R., Nutter F., Brown H.K., Vasiliadou I., Ottewell P.D., Holen I. (2013). Macrophages as potential targets for zoledronic acid outside the skeleton - Evidence from in vitro and in vivo models. Cell. Oncol..

[127] Prockop D.J. (2013). Concise review: two negative feedback loops place mesenchymal stem/stromal cells at the center of early regulators of inflammation. Stem Cells.

[128] Elchaninov A., Fatkhudinov T., Usman N., Arutyunyan I., Makarov A., Lokhonina A., Eremina I., Surovtsev V., Goldshtein D., Bolshakova G. (2018). Multipotent stromal cells stimulate liver regeneration by influencing the macrophage polarization in rat. World J. Hepatol..

[129] Lee S., Kivimäe S., Dolor A., Szoka F.C. (2016). Macrophage-based cell therapies: The long and winding road. J. Control. Release.

[130] Terai S., Tsuchiya A. (2017). Status of and candidates for cell therapy in liver cirrhosis: Overcoming the “point of no return” in advanced liver cirrhosis. J. Gastroenterol..

[131] Thomas J.A., Pope C., Wojtacha D., Robson A.J., Gordon-Walker T.T., Hartland S., Ramachandran P., Van Deemter M., Hume D.A., Iredale J.P. (2011). Macrophage therapy for murine liver fibrosis recruits host effector cells improving fibrosis, regeneration, and function. Hepatology.

[132] Ma P.-F., Gao C.-C., Yi J., Zhao J.-L., Liang S.-Q., Zhao Y., Ye Y.-C., Bai J., Zheng Q.-J., Dou K.-F. (2017). Cytotherapy with M1-polarized macrophages ameliorates liver fibrosis by modulating immune microenvironment in mice. J. Hepatol..

[133] Wan J., Benkdane M., Teixeira-Clerc F., Bonnafous S., Louvet A., Lafdil F., Pecker F., Tran A., Gual P., Mallat A. (2014). M2 Kupffer cells promote M1 Kupffer cell apoptosis: A protective mechanism against alcoholic and nonalcoholic fatty liver disease. Hepatology.

[134] Xu L., Yang F., Lin R., Han C., Liu J., Ding Z. (2014). Induction of M2 polarization in primary culture liver macrophages from rats with acute pancreatitis. PLoS ONE.

[135] Devey L., Ferenbach D., Mohr E., Sangster K., Bellamy C.O., Hughes J., Wigmore S.J. (2009). Tissue-resident macrophages protect the liver from ischemia reperfusion injury via a heme oxygenase-1-dependent mechanism. Mol. Ther..

[136] Preisser L., Miot C., Le Guillou-Guillemette H., Beaumont E., Foucher E.D., Garo E., Blanchard S., Frémaux I., Croué A., Fouchard I. (2014). IL-34 and macrophage colony-stimulating factor are overexpressed in hepatitis C virus fibrosis and induce profibrotic macrophages that promote collagen synthesis by hepatic stellate cells. Hepatology.

[137] Qin N., Yang F., Li A., Prifti E., Chen Y., Shao L., Guo J., Le Chatelier E., Yao J., Wu L. (2014). Alterations of the human gut microbiome in liver cirrhosis. Nature.

[138] Mazagova M., Wang L., Anfora A.T., Wissmueller M., Lesley S.A., Miyamoto Y., Eckmann L., Dhungana S., Pathmasiri W., Sumner S. (2015). Commensal microbiota is hepatoprotective and prevents liver fibrosis in mice. FASEB J..

[139] Zhang W., Gan D., Jian J., Huang C., Luo F., Wan S., Jiang M., Wan Y., Wang A., Li B. (2019). Protective Effect of Ursolic Acid on the Intestinal Mucosal Barrier in a Rat Model of Liver Fibrosis. Front. Physiol..

[140] Li X., Yao W., Yuan Y., Chen P., Li B., Li J., Chu R., Song H., Xie D., Jiang X. (2017). Targeting of tumour-infiltrating macrophages via CCL2/CCR2 signalling as a therapeutic strategy against hepatocellular carcinoma. Gut.

[141] Dong P., Ma L., Liu L., Zhao G., Zhang S., Dong L., Xue R., Chen S. (2016). CD86^+^/CD206^+^, Diametrically Polarized Tumor-Associated Macrophages, Predict Hepatocellular Carcinoma Patient Prognosis. Int. J. Mol. Sci..

[142] Raggi C., Correnti M., Sica A., Andersen J.B., Cardinale V., Alvaro D., Chiorino G., Forti E., Glaser S., Alpini G. (2017). Cholangiocarcinoma stem-like subset shapes tumor-initiating niche by educating associated macrophages. J. Hepatol..

[143] Kazankov K., Barrera F., Møller H.J., Rosso C., Bugianesi E., David E., Younes R., Esmaili S., Eslam M., McLeod D. (2016). The macrophage activation marker sCD163 is associated with morphological disease stages in patients with non-alcoholic fatty liver disease. Liver Int..

[144] Kong L.-Q., Zhu X.-D., Xu H.-X., Zhang J.-B., Lu L., Wang W.-Q., Zhang Q.-B., Wu W.-Z., Wang L., Fan J. (2013). The clinical significance of the CD163+ and CD68+ macrophages in patients with hepatocellular carcinoma. PLoS ONE.

